# Cost-effectiveness of a community-based cardiovascular disease prevention intervention in medically underserved rural areas

**DOI:** 10.1186/s12913-019-4117-y

**Published:** 2019-05-16

**Authors:** Hua Wang, Donald Kenkel, Meredith L. Graham, Lynn C. Paul, Sara C. Folta, Miriam E. Nelson, David Strogatz, Rebecca A. Seguin

**Affiliations:** 1 Cornell University, 292 Martha Van Rensselaer Hall, Ithaca, NY 14853 USA; 2 Cornell University, 2310 Martha Van Rensselaer Hall, Ithaca, NY 14853 USA; 3 Cornell University, 414 Savage Hall, Ithaca, NY 14853 USA; 4 Montana State University, 322 Reid Hall, Bozeman, MT 59717 USA; 5 Tufts University, 150 Harrison Ave, Boston, MA 02111 USA; 6 Hampshire College, 893 West St, Amherst, MA 01002 USA; 7 Bassett Research Institute, One Atwell Rd, Cooperstown, NY 13326 USA; 8 Cornell University, 412 Savage Hall, Ithaca, NY 14853 USA

**Keywords:** Cost-effectiveness analysis, Cardiovascular disease prevention, Rural, Economic evaluation, Quality adjusted life years, Women

## Abstract

**Background:**

Rural women experience health disparities in terms of cardiovascular disease (CVD) risk compared to urban women. Cost-effective CVD-prevention programs are needed for this population. The objective of this study was to conduct cost analysis and cost-effectiveness analyses (CEAs) of the *Strong Hearts, Healthy Communities (SHHC)* program compared to a control program in terms of change in CVD risk factors, including body weight and quality-adjusted life years (QALYs).

**Methods:**

Sixteen medically underserved rural towns in Montana and New York were randomly assigned to SHHC, a six-month twice-weekly experiential learning lifestyle program focused predominantly on diet and physical activity behaviors (n = 101), or a monthly healthy lifestyle education-only control program (n = 93). Females who were sedentary, overweight or obese, and aged 40 years or older were enrolled. The cost analysis calculated the total and per participant resource costs as well as participants’ costs for the SHHC and control programs. In the intermediate health outcomes CEAs, the incremental costs were compared to the incremental changes in the outcomes. The QALY CEA compares the incremental costs and effectiveness of a national SHHC intervention for a hypothetical cohort of 2.2 million women compared to the status quo alternative.

**Results:**

The resource cost of SHHC was $775 per participant. The incremental cost-effectiveness ratios from the payer’s perspective was $360 per kg of weight loss. Over a 10-year time horizon, to avert per QALY lost SHHC is estimated to cost $238,271 from the societal perspective, but only $62,646 from the healthcare sector perspective. Probabilistic sensitivity analyses show considerable uncertainty in the estimated incremental cost-effectiveness ratios.

**Conclusions:**

A national SHHC intervention is likely to be cost-effective at willingness-to-pay thresholds based on guidelines for federal regulatory impact analysis, but may not be at commonly used lower threshold values. However, it is possible that program costs in rural areas are higher than previously studied programs in more urban areas, due to a lack of staff and physical activity resources as well as  availability for partnerships with existing organizations.

**Trial registration:**

ClinicalTrials.gov identifier NCT02499731, registered on July 16, 2015.

## Background

Cardiovascular disease (CVD) accounts for about one-third of all deaths in the U.S. and is the leading cause of mortality [1]. Rural populations face CVD-related health disparities compared to their urban counterparts; they are less likely to meet physical activity recommendations and more likely to smoke, be overweight, and have type 2 diabetes [2, 3]. Rural women face additional health risk factors in terms of income, education, age, and insurance [2]. Environmental aspects of rural areas, including limited access to physical activity opportunities, healthy foods, and healthcare resources, contribute to development of these risk factors [4–6]. Therefore, women in rural, medically underserved areas are a critical population for CVD prevention interventions.

Health policy makers face difficult choices between funding targeted CVD prevention efforts, other public health efforts, and clinical healthcare. Economic evaluation methods are used to guide scarce societal resources to their highly valued use in improving health [7, 8]. Investments in prevention can be particularly attractive when they reduce the need for future clinical healthcare spending [9]. More often, prevention adds to healthcare costs, but can still provide an attractive return on investment compared to other options to improve health [10].

Community-based CVD, type 2 diabetes, and obesity prevention programs are generally found to be cost-effective [11–13] Because midlife and older rural women face health disparities and rural areas may lack healthy lifestyle resources, CVD prevention programs are critical for this population. Relatively few cost-effectiveness analyses (CEAs) have been performed for community-based healthy lifestyle interventions in rural areas [14–18]. Some of these previous studies were not randomized controlled trials [14, 18], combined results from urban and rural areas [14], had under 50 participants [17, 18] or did not report the number of participants [14], included men and women [14, 15, 18], and/or included young adults, as well as midlife and older adults [15, 17]. Only one previous CEA has been done on a community-based healthy lifestyle behavior change program studying only midlife and older rural women, in the United States, with over 50 participants [16].

In this paper, we report the results of economic evaluations of a rural community-based CVD prevention program targeted at overweight and obese rural women aged 40 and older. Strong Hearts, Healthy Communities (SHHC) was an innovative community-based six-month intervention informed by the socioecological framework to target key behaviors related to CVD prevention and overweight/obesity. We hypothesized that the cost-effectiveness of SHHC would compare favorably with other interventions and commonly used thresholds for acceptable costs per quality-adjusted life year (QALY) saved.

## Methods

### Research design

We conducted economic evaluations of SHHC alongside a two-armed randomized controlled efficacy trial. The economic evaluations include a program cost analysis (CA) and CEAs that adopted alternative perspectives and used multiple health outcome measures. The study protocol for the efficacy trial has been previously published [19]. The study was approved by the Cornell University and Bassett Healthcare Institutional Review Boards.

The efficacy trial compared the multilevel SHHC intervention to a minimal, education-only control program, Strong Hearts, Healthy Women (CON). Randomization occurred at the town level, with half randomized to deliver the SHHC intervention program and half to deliver the CON program. The primary outcomes of the efficacy trial were kg of body weight and BMI; physiological measures and two composite measures of cardiovascular disease risk were also evaluated [20].

### Participants and setting

Sedentary overweight or obese women aged 40 and older were recruited from 16 medically underserved rural towns in Montana and New York. Participants were recruited by local health educators. Eligible participants were female, 40 years or older, overweight (BMI > 25), sedentary, English-speaking, and had their physician’s approval to participate. Participants with blood pressure > 160 (systolic) or > 100 (diastolic), heart rates of < 60 or > 100, or cognitive impairments were excluded. All participants provided written informed consent. A total of 436 participants were assessed for eligibility; 194 participants enrolled.

### Interventions

Based on previous effective programs [21–23], the SHHC multilevel CVD prevention program targeted individual (experiential learning around dietary intake and physical activity, including aerobic exercise and strength training), social (activities including family and friends), and community (civic engagement curriculum designed to catalyze positive built environment) levels. SHHC classes met for 1 hour twice weekly for 24 weeks (48 classes total).

The Strong Hearts, Healthy Women control (CON) classes served as the reduced-dose, education-only, minimal intervention control program. Classes provided evidence-based healthy lifestyle information (e.g. current dietary and physical activity guidelines) presented didactically. Participants did not engage in physical activity, skill building, or other active learning elements (e.g. reflection, monitoring) or civic engagement during the class sessions. The CON classes met for a one-hour class once per month over 24 weeks (six classes total).

### Perspectives of the CEA

The CA and one set of CEAs were conducted from the payer perspective, which means we estimated how much the intervention’s payer or sponsor paid for the intervention. When we adopted the payer perspective, we focused on costs directly incurred to administer and implement the program. The results of the payer perspective analysis provide crucial information for local health policy makers to judge whether and under what circumstances SHHC should be disseminated.

As recommended by the Second Panel on Cost-Effectiveness in Health and Medicine [7], we also conducted Reference Case CEAs from two broader perspectives. The Reference Case CEAs from the broad societal perspective consider all significant health outcomes and costs, including participants’ direct and opportunity costs, that flow from the intervention. The Reference Case CEAs from the healthcare sector perspective consider formal healthcare sector (medical) costs borne by third-party payors or paid out-of-pocket by patients. The results of the Reference Case analyses allows comparison of the cost-effectiveness of SHHC to existing cost-effectiveness research on a broad range of prevention and clinical interventions.

### Health outcomes

In one set of CEAs, we used the health outcomes measured in the efficacy trial. In these CEAs we examined the cost per kg of body weight reduction; the cost per point of BMI reduction; the cost per mg/L of C-reactive protein (CRP) reduction; and the cost per point of Simple 7 increase. Simple 7 is a composite cardiovascular health metric composed of four health behaviors (non-smoking, appropriate BMI, physical activity, healthy diet) and three health factors (total cholesterol, blood pressure, fasting glucose) [24].

In another CEA we used the ten-year risk for atherosclerotic cardiovascular disease (ASCVD) to conduct CEA in terms of cost per QALY saved. ASCVD risk was calculated using the Pooled Cohort Equations based on age, total cholesterol, high-density lipoprotein cholesterol, systolic blood pressure (including treated or untreated status), diabetes, and current smoking status [25]. We used previously published estimates to calculate the QALYs saved per ASCVD event prevented [26].

### Costs

For the CA and the CEAs from the payer perspective, we identified the resources directly used in program administration and implementation and collected measures of the associated tangible costs. The categories of resource use were labor, facilities (space and utilities), food, equipment, curriculum printing, and other. Labor resources include staff training and preparation time, as well as staff time to deliver the interventions. Information on resource use was provided as administrative records or collected via surveys of the program administrators. Administrative records on staff compensation including salary and fringe benefits were used to calculate labor costs. Administrative records also provided the information on the costs of food, equipment, and curriculum printing. The labor and food costs are provided at the site-level. The equipment and printing costs are overall estimates. The space rental fees and other costs are estimated based on a dataset from a survey of agents and coordinators. Site level costs are taken from administrative records (labor, food, equipment, and printing) or estimated as site level means from surveys (rent, other costs, and participants’ gasoline and time). Missing site level costs for labor, rent, and others are replaced by the mean cost per site within the SHHC or CON program. Missing food and participant costs are estimated based on the mean cost per enrollee per site within program. The total costs for SHHC and CON are the sum of all their sites’ costs.

For the Reference Case CEAs from the societal perspective, we measured not only the direct program resource costs, but also the opportunity cost of all resources used as a result of the intervention. Costs to participants are an important component of the opportunity costs included from the societal perspective. Participants give up time that could have been used in other valued ways such as labor market work, household work, or leisure activities. We collected information on participant costs from surveys of participants. We followed standard practice and measured the value of participants’ time based on the relevant wage rates.

For the Reference Case CEAs from the healthcare sector perspective, we used estimates of the medical costs of CVD events [27]. The estimates are from a study that used administrative claims data from a large U.S. health plan to predict medical costs of coronary heart disease and stroke events.

### Analyses

The CA calculated the total and per participant costs of the resources used in the administration and implementation of the SHHC and CON interventions. The CEAs calculate incremental cost-effectiveness ratios (ICERs) as the ratio of the incremental costs over the incremental effectiveness.

In the intermediate health outcomes CEAs, the incremental costs were calculated as the per participant costs in the SHHC intervention minus the per participant costs in the CON. The incremental effectiveness estimates were taken from the between group multivariate analysis of the SHHC intervention’s impact compared to the CON on weight, BMI, CRP, and Simple 7 score [20].

In the QALY CEA, the incremental costs and effectiveness compare the SHHC intervention to the status quo alternative (no intervention). We conducted the analysis for a hypothetical cohort of 2.2 million women. The cohort size corresponds to a hypothetical nationwide SHHC intervention that reaches all midlife and older overweight and obese women in rural medically underserved communities [28]. We used the incidence rates for 55–64 year-old women to predict the status quo number of CVD events in a cohort this size over a 10-year time horizon. We predicted the number of CVD events after the hypothetical SHHC intervention based on the pre-post within group multivariate analysis of the SHHC’s intervention impact on ASCVD risk (Table 2 in [20]). We use the pre-post within group analysis because the estimated  between-group change for ASCVD risk (Table 3 in [20]) has a wider confidence interval possibly due to a relatively small sample size, less suitable controls, or other factors. For each CVD event, we calculated the associated QALYs and healthcare costs based on previously published estimates [26, 27]. The QALY losses from CVD events were calculated relative to the expected QALYs in a population of older obese women.

We conducted probabilistic sensitivity analysis to characterize parameter uncertainty in the CEAs. The analysis treats each parameter as a random variable with an assumed mean, range, and probability distribution. We conducted Monte Carlo simulations with a sample of 1000 observations, each observation representing a hypothetical trial. The simulation results are 1000 observations of incremental costs, effects, and ICERs. We graphically present the results on cost-effectiveness planes.

For the QALY CEA, we use cost-effectiveness acceptability curves to present the probability that the SHHC intervention is acceptable for a range of willingness-to-pay thresholds. The range of willingness-to-pay thresholds for QALYs includes consensus estimates of societal willingness-to-pay per QALY saved Analyses were conducted using Stata 15 (StataCorp. 2017. *Stata Statistical Software: Release 15*. College Station, TX: StataCorp LLC).

## Results

### Program cost analysis

The total costs of the direct resources used in the administration and implementation of the SHHC intervention were $78,229 (Table 1, all costs are in 2016 US dollars).

**Table 1:** Program resource costs from the payer perspective, $

	SHHC	CON
Cost category		
Labor	35,238	8563
Space	2291	286
Food	7182	995
Equipment	27,781	0
Printing	1336	195
Other	4400	0
Total cost	78,229	10,040
Number of participants	101	93
Total cost per person	775	108

The resource cost per participant was $775. The total costs of the minimal intervention education-only CON were $10,040 and the cost per participant was $108. From the societal perspective that includes participants’ direct and opportunity costs, the cost per SHHC participant increases to $1087 and the cost per CON participant increases to $201 (Table 2). The largest cost component is the opportunity cost of participants’ time. The Second Panel on Cost-Effectiveness in Health and Medicine [7] recommends including the opportunity costs of time, but a previous CEA of a similar intervention was not able to take them into account [16, 29, 30]. These results in Table 2 highlight the importance of the opportunity cost when evaluating participant-time intensive interventions.

**Table 2:** Participants’ direct and opportunity costs, $ per person

	SHHC	CON
Cost category		
Gasoline for travel in personal auto	30	17
Time costs	1058	183
Total participant cost	1087	201

### Intermediate health outcomes CEAs

The calculated ICERs from the payer perspective show that the SHHC intervention costs $360 per kg of weight loss, $939 per unit of BMI reduction, $580 per mg/L CRP reduction, and $995 per unit increase in Simple 7 (Table 3). The ICERs from the societal perspective are $840 per kg of weight loss, $2187 per unit of BMI reduction, $1351 per mg/L CRP reduction, and $2318 per unit increase in Simple 7.

**Table 3:** Cost-effectiveness of SHHC for intermediate health outcomes

	Incremental between	ICER from the perspective of	
	SHHC–CON	Payer	Society
Costs, $ per participant			
Resource	667	√	√
Participant	887		√
Total	1553		
Outcomes, per participant			
Weight loss, kg	−1.85	360	840
BMI reduction	−0.71	939	2187
CRP reduction, mg/L	−1.15	580	1351
Simple 7 increase	0.67	995	2318

The “√” indicates incremental costs used in the ICER calculation under alternative perspectives

**Fig. 1:**
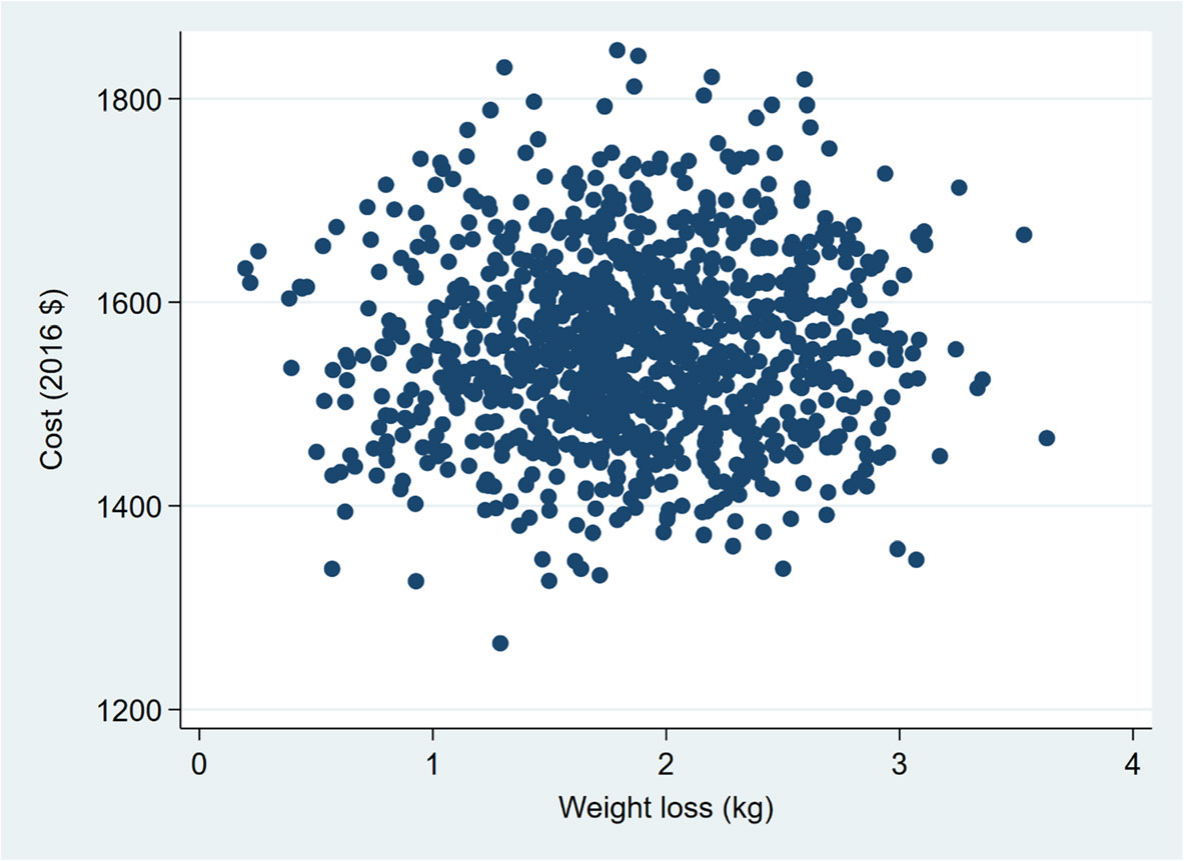
Sensitivity analysis of SHHC’s cost and effectiveness on weight loss

**Fig. 2:**
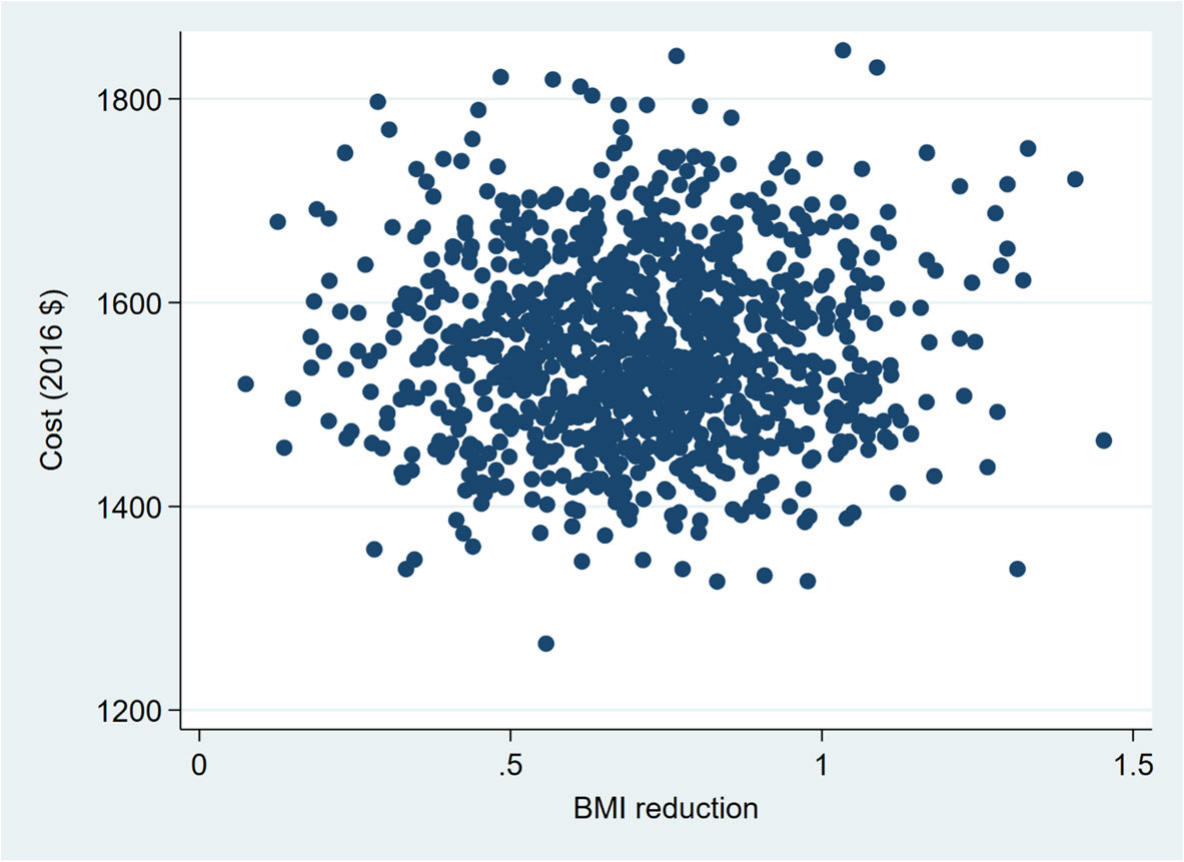
Sensitivity analysis of SHHC’s cost and effectiveness on BMI reduction

**Fig. 3:**
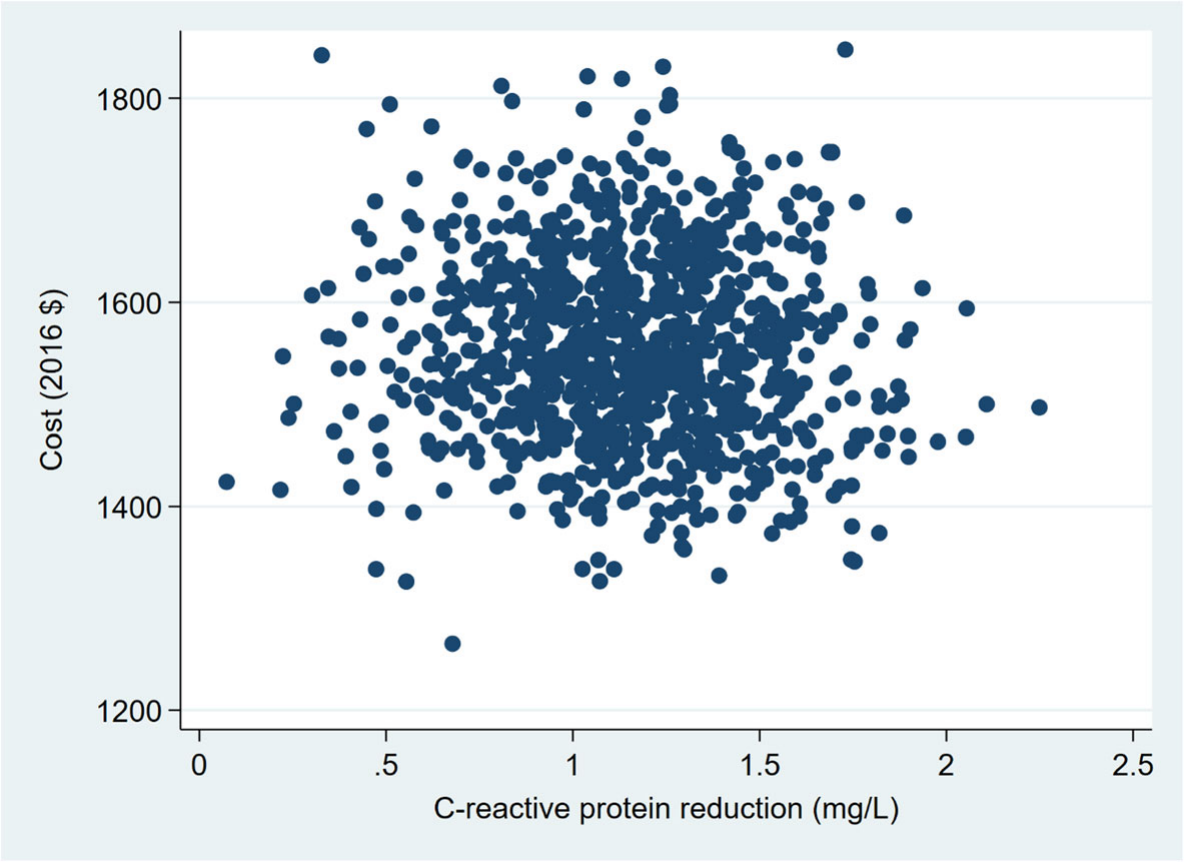
Sensitivity analysis of SHHC’s cost and effectiveness on CRP reduction

**Fig. 4:**
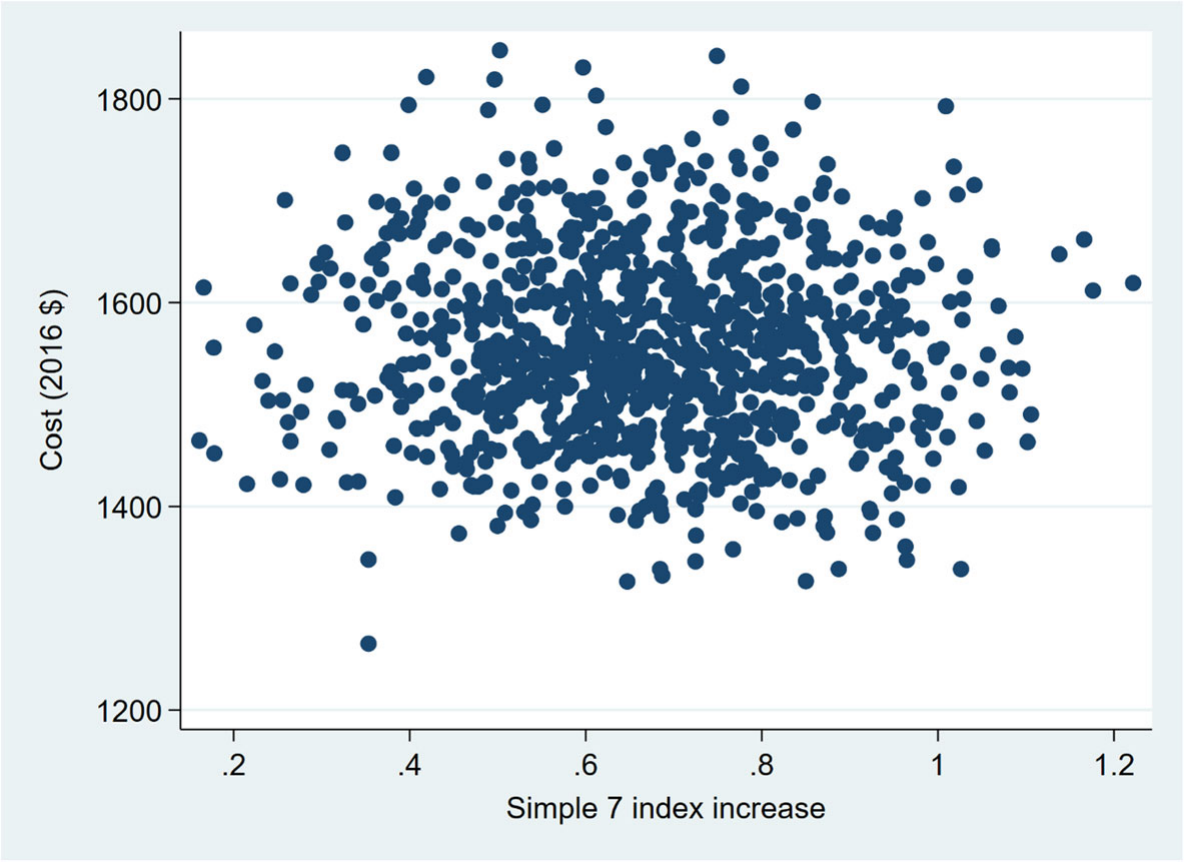
Sensitivity analysis of SHHC’s cost and effectiveness on Simple 7 increase

The variables examined in the probabilistic sensitivity analysis are presented in Tables 4 and 5. The probabilistic sensitivity analysis show that there is considerable uncertainty in the estimated ICERs for the intermediate health outcomes (Figs. 1, 2, 3 and 4). The width (on the X axis) of the scatter plots of points in Figs. 1, 2, 3 and 4 reflects uncertainty about the SHHC intervention’s effectiveness (incremental changes in the outcomes), based on the 95% confidence intervals of the estimates from the between group multivariate analysis in the efficacy trial [20]. The height (on the Y axis) of the scatter plots of points reflects uncertainty about SHHC costs (incremental changes in costs from the societal perspective). To illustrate the degree of uncertainty in the resulting ICERs for weight loss, the 5 and 95% ICERs are $548 and $1805 per kg of weight loss. Note that in Table 5, the incidence rates for CHD and stroke are not the assumed CHD or stroke rates for the hypothetical population in the probabilistic sensitivity analysis. The ratio of the two incidence rates is used in the probabilistic sensitivity analysis to partition the reduction of an ASCVD event into a CHD event reduction and a stroke event reduction.

**Table 4:** Parameters and variables examined in the probabilistic sensitivity analysis. Parameters with fixed values

Parameter	Value
N (trials)	1000
Discount rate	0.03
Incidence rate, 55–64 Females	
CHD	0.003
Stroke	0.002

**Table 5:** Parameters and variables examined in the probabilistic sensitivity analysis. Parameters with varied values

Parameter	Distribution	Mean	Range			
Outcomes						
BMI reduction	Normal	0.71	0.08	–	1.35	(95% CI of mean)
Weight loss	Normal	1.85	0.16	–	3.55	(95% CI of mean)
CRP reduction, mg/L	Normal	1.15	0.15	–	2.16	(95% CI of mean)
Simple 7 increase	Normal	0.67	0.14	–	1.21	(95% CI of mean)
Annual ASCVD risk reduction	Log normal	0.00096	0.00043	–	0.00149	(95% CI of mean)
Costs, per person						
Resources of SHHC	Υ, gamma	775	581	–	968	(±25% of mean)
Resources of SHHC-CON	Υ, gamma	667	500	–	833	(±25% of mean)
Participant of SHHC	Υ, gamma	1087	815	–	1359	(±25% of mean)
Participant of SHHC-CON	Υ, gamma	887	665	–	1108	(±25% of mean)
Medical cost, 2013 $						
CHD (nonfatal mi)	Υ, gamma	62,200	52,870	–	71,530	(±15% of mean)
Stroke (nonfatal)	Υ, gamma	20,509	17,433	–	23,585	(±15% of mean)
Follow-up visit, 2013 $	Υ, gamma	75	53	–	98	(±30% of mean)
QALYs						
Women age 55–64, BMI 30–40	β, beta	0.798	0.678	–	0.918	(±15% of mean)
CHD	β, beta	0.697	0.592	–	0.802	(±15% of mean)
Stroke	β, beta	0.590	0.502	–	0.679	(±15% of mean)

The incidence rates are not the assumed CHD or stroke rates for the hypothetical population in the probabilistic sensitivity analysis. The ratio of the two incidence rates is used in the probabilistic sensitivity analysis to partition the reduction of an ASCVD event into a CHD event reduction and a stroke event reduction

### QALY CEA

From the societal perspective, which reflects all costs of the intervention to society over a 10-year time horizon, a hypothetical national SHHC is estimated to cost $238,271 per QALY gained (Table 6). From the healthcare sector perspective, the national SHHC is estimated to cost $62,646 per QALY.

**Table 6:** Costs and effectiveness over the next 10 years of SHHC for a national sample

	SHHC	Status Quo	Incremental of	ICERs from perspective of	
			SHHC−Status Quo	*Healthcare*	*Society*
Costs, $					
Resource	1,716,954,549	0	1,716,954,549	√	√
Healthcare	3,608,071,971	4,465,435,607	−857,363,637	√	√
Participant	2,409,841,516	0	2,409,841,516		√
Total	7,734,868,036	4,465,435,607	3,269,432,429		
Health effects					
Heart events	53,734	66,502	−12,768		
Stroke events	35,823	44,335	−8512		
QALYs saved	15,031,833	15,018,112	13,721	62,646	238,271

“√” indicates incremental costs used in ICER calculation under alternative perspectives. The heart events and stroke events are non-fatal events

The hypothetical national SHHC is estimated to prevent 12,768 heart events and 8512 stroke events (both non-fatal events) (Table 6). By preventing these events, SHHC is estimated to reduce healthcare sector costs by $857 million, which is the healthcare cost savings that would have been spent to treat the heart and stroke events in absence of the national SHHC. However, these healthcare cost savings are not sufficient to offset the resource and participant costs (about $4127 million) of the national intervention to a hypothetical cohort of 2.2 million women.

**Fig. 5:**
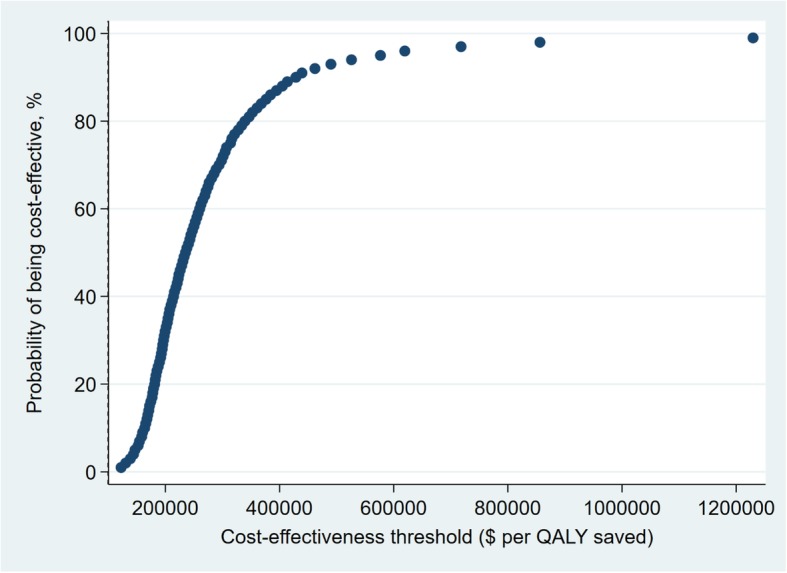
Cost-effectiveness acceptability curve based on a sensitivity analysis of SHHC’s cost and ASCVD risk reduction

The probabilistic sensitivity analysis shows that there is again considerable uncertainty in the estimated societal cost per QALY gained. The results are summarized in a CEA acceptability curve, which shows the probabilities the national SHHC intervention is cost-effective at different thresholds for societal willingness-to-pay per QALY (Fig. 5). The probabilities of cost-effectiveness are measured as the fraction of estimated ICERs in the sensitivity analysis that are below each threshold.

There is not agreement on the appropriate willingness-to-pay threshold [8], but thresholds of $50,000 and $100,000 per QALY are commonly considered [29]. The national SHHC is very unlikely to be cost-effective at these thresholds. A recent federal guideline for regulatory impact analysis estimates willingness-to-pay per QALY based on estimates of the value of a statistical life (VSL) [30]. The range of VSL estimates imply that willingness-to-pay ranges from $230,000 to $750,000 per QALY; the central VSL estimate implies willingness-to-pay per QALY is $490,000. The probability the national SHHC intervention is cost-effective is 48% using the $230,000 threshold, 93% using the central threshold of $490,000 and almost 97% using the $750,000 threshold.

### Site-level cost-effectiveness analysis

In addition to the overall CA and CEA of the SHHC program, which took place at eight SHHC sites (towns) and eight CON sites in Montana and New York, we estimated SHHC’s costs and cost-effectiveness at the site level. The site level CA is the first step of the CA for the SHHC and CON interventions as a whole. The overall costs for SHHC and CON are simply the sum of the estimated costs in the eight SHHC sites and eight CON sites, respectively. Results of the site level CA are reported in Appendix Tables 9 and 10. The primary purpose of the site level CEA is to investigate the variation of SHHC’s cost-effectiveness measured by ICERs. Unlike the overall CEA for the intermediate health outcomes (BMI, etc.), which compared SHHC with the CON, the site level CEA looked at incremental changes within group for SHHC sites (compared SHHC with status quo or the post- versus pre-intervention) for all outcomes. The site level effects of SHHC are reported in Appendix Table 11. The cost-effectiveness of SHHC in terms of ICERs is estimated from both the payer and society perspective and reported in Tables 7 and 8, respectively.

**Table 7:** By site - Cost-effectiveness of SHHC. ICERs (vs Status Quo) from the payer or healthcare sector perspective

Site ID	Weight loss	BMI reduction	CRP reduction	Simple 7 increase	ASCVD QALY saved
3	284	759	*93,477	3495	136,276
5	156	394	442	675	455,779
6	*830	*2502	482	1341	58,924
8	343	954	921	921	186,004
11	157	625	341	1811	−16,991
12	793	1920	420	370	396,841
14	321	829	471	439	− 9480
16	290	741	572	859	*1,386,823

ICERs for QALY saved are estimated from the healthcare perspective and ICERs for other outcomes are from the payer perspective. The negative ICERs represent cost savings. The “*” implies that the effect is in the unexpected direction. For example, an ICER for weight loss with “*” implies that on average participants gained instead of lost body weight after the SHHC

**Table 8:** By site - Cost-effectiveness of SHHC. ICERs (vs Status Quo) from the societal perspective

Site ID	Weight loss	BMI reduction	CRP reduction	Simple 7 increase	ASCVD QALY saved
3	683	1823	*224,455	8392	414,770
5	408	1028	1153	1762	1,290,021
6	*1692	*5099	983	2732	184,905
8	681	1892	1827	1827	430,353
11	436	1729	945	5012	63,429
12	2291	5547	1214	1068	1,264,574
14	719	1854	1055	983	56,118
16	790	2022	1560	2342	*3,674,482

The “*” implies that the effect is in the unexpected direction. For example, an ICER for weight loss with “*” implies that on average participants gained instead of lost body weight after the SHHC

As in the sensitivity analysis results, the site level analysis results also show considerable variation in SHHC’s costs, effects, and cost-effectiveness across sites. For example, the total resource cost per participant across SHHC sites ranges from $575 (Site 12) up to $1106 (Site 8) (Appendix Table 10). The change of health outcome may be in the opposite of the expected direction for all health outcomes except the Simple 7. For example, SHHC participants from Site 6 on average gained weight (instead of lost) by 1.13 kg after the intervention and SHHC participants from Site 16 on average had a very small increase of ASCVD risk over the next 10 years by 0.07%, which results in a large ICER (3.67 million from societal perspective) for per QALY lost (Appendix Table 11). While from the societal perspective ICERs for QALY saved are relatively high in most sites, from the healthcare perspective, they are negative, indicating cost savings in Site 11 and Site 14 (Tables 7 and 8). In these two sites the SHHC resource cost is below the medical care cost that would be saved or prevented over the next 10 years per QALY saved.

## Discussion

In the CA, the resource cost per SHHC participant ($775) was somewhat higher than the costs of a few other previously studied weight loss and nutrition interventions. The average cost of providing WISEWOMAN services was $270 per participant [31]. Because data was compiled from unique WISEWOMAN programs in multiple states, it is not possible to directly compare the program to SHHC (e.g. number of classes). An economic evaluation of the Lifestyle Education for Activity and Nutrition (LEAN) Study reported average cost per participant ranging from $54 (multi-sensor armband only) to $365 (multi-sensor armband plus 14 group weight loss education classes) [32]. The only previous CEA of a community-based healthy lifestyle program in the United States with midlife and older rural women, having over 50 participants was a 12-month extended care lifestyle maintenance program after an initial six-month weight loss program [16]. Participants attended twice-monthly (24 total) face-to-face group sessions or twice-monthly (24 total) individual telephone sessions or received 24 newsletters [16]. Program costs were $420, $268, and $226 per participant for the face-to-face, telephone, and control programs, respectively [16]. SHHC cost $775 per participant for the 48 sessions and the 6-session control program cost $108 per participant. Conversely, SHHC is comparable in cost per participant to an economic evaluation of the Expanded Food and Nutrition Education Program, which reported average direct (resource) costs per graduate as $715 [33]. In this program, participants attended six or more nutrition education classes, compared to 48 classes for SHHC participants.

Adding the opportunity cost of participants’ time substantially increases the costs from the societal perspective. Program administrators considering adoption of SHHC do not necessarily consider these costs because they do not have a direct impact on program budgets. However, the need to consider these costs imposed on participants is well-established in the theoretical foundations of CEA. Moreover, time costs might be an important barrier to widespread participation in an intensive intervention like SHHC.

Regarding ASCVD risk reduction, the cost-effectiveness of SHHC may be compared with the WISEWOMAN program [31, 34]. A cost-effectiveness study of the WISEWOMAN found that the program cost $4400 (about $5300 in 2016 dollars) per life-year gained. Calculated in a similar way, SHHC would cost about $8600 per life-year gained. The higher cost per life-year gained in SHHC than WISEWOMAN may be related to two factors. One is that the SHHC intervention is more intensive and therefore, more costly. The other factor is the difference in sample characteristics. Compared with the WISEWOMAN sample, SHHC participants are older (average age 59 versus 52) and are already almost a completely non-smoking group (5% versus 23% smoking). The sample difference suggests that it may be more difficult for the SHHC sample to achieve ASCVD risk improvement quickly than the WISEWOMAN sample.

By the measure of cost per QALY (or disability -adjusted life year (DALY)) saved, SHHC is less cost-effective compared with other lifestyle interventions that also help older people to lose weight [12, 13]. Seven such studies included in two recent review studies report cost-effectiveness ratios ranging from about 3700US$ (4-year time frame) per QALY to 92,100–99,200US$ (12-month time frame) per DALY from the healthcare sector perspective [35–39]. One study reported about 13,700–15,300US$ (6-month time frame) per life-year gained from the society perspective [40]; and another study reported about 51,700US$ (12-month time frame) per QALY with an unclear study perspective [41]. Besides SHHC’s high intensiveness and disadvantaged target population, its high estimated cost per QALY may be related to the parameters and approach used in the estimation. Other studies often use a longer time frame. We currently estimate ASCVD risk improvement for the next 10 years. If we assume the same effect on ASCVD risk improvement would continue for another 10 years (from continued lower weight, etc.), the SHHC’s cost per QALY would be about $68,700 from the societal perspective and $5300 from the health sector perspective.

It is unknown whether CVD prevention programs similar to SHHC cost more to implement in rural versus urban areas. Urban communities are more likely to have public recreational facilities and programs [42] and may have more opportunities to form cooperative agreements and partnerships to maximize the use of facilities and staff time (e.g. with fitness centers or community organizations), reducing the overall cost of a CVD prevention program similar to SHHC.

A national SHHC intervention is likely to be cost-effective at willingness-to-pay thresholds based on guidelines for federal regulatory impact analysis. However, it is not likely to be cost-effective at commonly used lower threshold values. The cost-effectiveness of SHHC reflects a common tradeoff in the economics of prevention. Each CVD event prevented generates substantial healthcare cost savings. However, the intervention must be delivered to a large number of participants per CVD event prevented, which can be particularly difficult in medically underserved rural areas.

### Strengths

This study is the first to evaluate the economic effectiveness of a multilevel community-based CVD prevention program for midlife and older women in rural, medically underserved areas via comprehensive CA and CEAs. Previous community-based lifestyle behavior change interventions in rural areas lacked comparison groups; had small sample sizes; and/or included younger adults, both men and women; or included telephone-based programs. Additionally, previous studies did not include participant time costs.

### Limitations

SHHC was a multilevel intervention, which required additional staff and participant time, and included social and community components that were not measured. Primary outcomes for SHHC were measured at six months; future CEAs should collect data that would enable measurement of the longer-term impact on individuals, as well as on social and community components targeted by SHHC.

One limitation of our study is not having converted all of the intervention benefits into QALYs, which can be used to compare with a wider range of interventions. The estimated QALYs saved in our analysis come from the reduced ASCVD risk by the SHHC program. Other benefits of the SHHC program may also save QALYs. For example, the SHHC program reduces BMI, which may prevent diabetes and save QALYs.

Our assumption that with the initial intervention cost of $775, the intervention’s benefit of ASCVD risk reduction would last for the next 10 years is not entirely reasonable due to the likelihood of weight regain or changes in other relevant factors. Additional costs may be incurred to maintain participants’ healthier status and ASCVD benefit but they may also increase the total intervention cost. Not taking into account the possible future costs is a limitation of this study.

We attribute the change in ASCVD risk scores pre and post the SHHC program as the causal effect of the intervention. This approach of using the prediction model for ASCVD risk score in a causal sense has limitations. This is a general problem with chronic disease prevention: it is costly and takes decades to run randomized controlled trials with hard ASCVD events as outcomes, so it is common to use epidemiologic modelling to translate the changes in outcomes measured in the randomized controlled trials into the changes in health outcomes and QALYs.

Another limitation of our study is not using the lifetime frame for the estimation of the benefits and costs of the reduced ASCVD risk by the SHHC program. We use the 10-year frame because the ASCVD risk measure is for a 10-year period. The limited time horizon may result in under- or over-estimated cost-effectiveness of the SHHC on ASCVD risk.

The last limitation of our study is that we do not have cost information for the ASCVD events for our study population. However, our estimates of ICERs for QALY saved are not very sensitive to the medical costs. For example, the ICERs for QALYs saved decrease slightly (more from the health sector perspective than from the societal perspective) if we increase the assumed stroke event cost from $20,509 to $50,000.

### Recommendations

To improve the cost-effectiveness of SHHC, the program could be adapted to achieve greater impacts on weight, Simple 7, and ASCVD as well as to further impact additional individuals (through individual, social, or community components). The intervention could also reduce staff from one educator and one program assistant down to just one educator. Participant time or other costs, such as space rental, could also be reduced. To impact more individuals, participants could attend classes with a friend or family member.

## Conclusions

The results of the economic evaluations of the SHHC intervention are informative for U.S. health policy. Policy makers should give a higher priority to implement other more cost-effective interventions, but the SHHC intervention still represents a reasonable return on investment. There might also be opportunities to better target the intervention to improve cost-effectiveness and it may be that programs similar to SHHC are more expensive to operate in rural areas.

## Appendix

**Table 9:** By site - Cost by categories, $

Group	Site ID	Enrollees	Resource cost						Participant cost	
			Food	Labor	Space	Other	Equipment	Printing	Gas	Time
CON	1	14	199	424	36	0	0	24	641	1496
CON	2	13	156	768	36	0	0	24	145	2096
CON	7	9	51	528	36	0	0	24	49	3004
CON	9	12	98	1772	36	0	0	24	82	1575
CON	10	11	148	1860	36	0	0	24	191	2016
CON	13	10	93	1070	36	0	0	24	173	1833
CON	15	12	125	1070	36	0	0	24	208	2199
CON	17	12	125	1070	36	0	0	24	208	2199
SHHC	3	13	992	4405	50	1000	3473	167	385	13,748
SHHC	5	13	1016	3424	150	550	3473	167	385	13,748
SHHC	6	12	959	5512	1150	0	3473	167	165	11,520
SHHC	8	12	1212	6440	475	1500	3473	167	355	12,690
SHHC	11	13	851	4288	50	0	3473	167	592	15,015
SHHC	12	12	306	2360	100	500	3473	167	355	12,690
SHHC	14	11	781	4405	286	550	3473	167	326	11,633
SHHC	16	15	1066	4405	30	300	3473	167	444	15,863

**Table 10:** By site - Total costs per participant, $

Group	Site ID	Resources	Resources and participant
CON	1	49	201
CON	2	76	248
CON	7	71	410
CON	9	161	299
CON	10	188	389
CON	13	122	323
CON	15	105	305
CON	17	105	305
SHHC	3	776	1863
SHHC	5	675	1762
SHHC	6	938	1912
SHHC	8	1106	2193
SHHC	11	679	1880
SHHC	12	575	1663
SHHC	14	878	1965
SHHC	16	629	1716

**Table 11:** By site - Effects of SHHC (vs Status Quo), per participant

Site ID	Weight, kg	BMI	CRP	Simple 7	ASCVD risk,%	QALYs	Medical cost,$
3	−2.73	−1.02	0.01	0.22	−0.61	0.0039	− 244
5	−4.32	−1.71	−1.53	1	−0.2	0.0013	−81
6	1.13	0.38	−1.95	0.7	−1.2	0.0077	− 483
8	−3.22	−1.16	−1.2	1.2	−0.69	0.0044	− 278
11	−4.32	−1.09	−1.99	0.38	−2.32	0.0149	− 933
12	−0.73	−0.3	− 1.37	1.56	− 0.19	0.0013	−78
14	−2.73	− 1.06	− 1.86	2	−2.57	0.0166	− 1035
16	−2.17	−0.85	− 1.1	0.73	0.07	−0.0005	30

Notes: Changes for ASCVD risk, QALYs saved, and Medical cost are for the next 10 years

## Abbreviations

ASCVD: Atherosclerotic cardiovascular disease
CA: Cost analysis
CEA: Cost-effectiveness analysis
CHD: Coronary heart disease
CON: Minimal, education-only control program
CRP: C-reactive protein
CVD: Cardiovascular disease
DALY: Disability-adjusted life year
ICER: Incremental cost-effectiveness ratio
LEAN: Lifestyle Education for Activity and Nutrition
QALY: Quality-adjusted life year
SHHC: Strong Hearts, Healthy Communities
VSL: Value of a statistical life
